# Community Rates of IgG4 Antibodies to *Ascaris* Haemoglobin Reflect Changes in Community Egg Loads Following Mass Drug Administration

**DOI:** 10.1371/journal.pntd.0004532

**Published:** 2016-03-18

**Authors:** Johnny Vlaminck, Taniawati Supali, Peter Geldhof, Cornelis H. Hokke, Peter U. Fischer, Gary J. Weil

**Affiliations:** 1 Infectious Diseases Division, Department of Internal Medicine, Washington University School of Medicine, St. Louis, Missouri, United States of America; 2 Department of Parasitology, Faculty of Medicine, University of Indonesia, Jakarta, Indonesia; 3 Laboratory for Parasitology, Department of Virology, Parasitology and Immunology, Ghent University, Merelbeke, Belgium; 4 Department of Parasitology, Center for Infectious Diseases, Leiden University Medical Center, Leiden, The Netherlands; Universidad Nacional Autónoma de México, MEXICO

## Abstract

**Background:**

Conventional diagnostic methods for human ascariasis are based on the detection of *Ascaris lumbricoides* eggs in stool samples. However, studies of ascariasis in pigs have shown that the prevalence and the number of eggs detected in the stool do not correlate well with exposure of the herd to the parasite. On the other hand, an ELISA test measuring antibodies to *Ascaris suum* haemoglobin (AsHb) has been shown to be useful for estimating transmission intensity on pig farms. In this study, we further characterized the AsHb antigen and screened samples from a population-based study conducted in an area that is endemic for *Ascaris lumbricoides* in Indonesia to assess changes in AsHb antibody rates and levels in humans following mass drug administration (MDA).

**Methodology/Principal findings:**

We developed and evaluated an ELISA to detect human IgG4 antibodies to AsHb. We tested 1066 plasma samples collected at different times from 599 subjects who lived in a village in rural Indonesia that was highly endemic for ascariasis. The community received 6 rounds of MDA for lymphatic filariasis with albendazole plus diethylcarbamazine between 2002 and 2007. While the AsHb antibody assay was not sensitive for detecting all individuals with *Ascaris* eggs in their stools, the percentage of seropositive individuals decreased rapidly following MDA. Reductions in antibody rates reflected decreased mean egg output per person both at the community level and in different age groups. Two years after the last round of MDA the community egg output and antibody prevalence rate were reduced by 81.6% and 78.9% respectively compared to baseline levels.

**Conclusion/Significance:**

IgG4 antibody levels to AsHb appear to reflect recent exposure to *Ascaris*. The antibody prevalence rate may be a useful indicator for *Ascaris* transmission intensity in communities that can be used to assess the impact of control measures on the force of transmission.

## Author Summary

Ascariasis is a neglected tropical disease caused by the intestinal nematode *Ascaris lumbricoides* that affects hundreds of millions of people in the developing world. Current methods for diagnosis of this infection are based on detecting eggs in the stool that are excreted by adult *Ascaris* worms. However, these methods have limited sensitivity for recent infections, and they do not detect infections with immature parasite stages that do not always result in the establishment of adult worms in the human intestine. We have previously shown that an assay for antibodies to *Ascaris* hemoglobin in pig serum is useful for assessing transmission of *Ascaris* infections on pig farms. In this study, we developed and evaluated a similar antibody assay that is based on the detection of human IgG4 antibodies to *Ascaris* haemoglobin (AsHb). Community antibody rates decreased rapidly following mass drug administration of the anthelmintic drug albendazole, and this decrease reflected reduced *Ascaris* egg excretion at the community level. This antibody test may be a useful tool for assessing the impact of control measures on the transmission of new *Ascaris* infections in endemic populations.

## Introduction

An estimated 1.45 billion people worldwide are infected with three soil transmitted helminths (STH) *Ascaris lumbricoides*, *Trichuris trichiura*, or hookworms [1]. Among the STH, infections with *A*. *lumbricoides* are most common and its public health impact is estimated to be approximately 1.31 million daily adjusted life years (DALYs) [1]. Current STH control programs are focused on morbidity control through community based deworming of school-aged children by annual or semiannual administration of a single dose of anthelmintics such as albendazole or mebendazole [2]. In addition, the large elimination programs for lymphatic filariasis and onchocerciasis provide anthelminthics to entire at risk populations that are also effective against *A*. *lumbricoides* [3, 4].

The intensity of *A*. *lumbricoides* infection is routinely measured by the number of eggs per gram (EPG) in stool by the Kato-Katz fecal thick-smear technique [5]. This method is useful for quickly assessing infection prevalence rates for evaluating the efficacy of control programs. However, the sensitivity of the Kato Katz smear is reduced in areas with low infection rates and intensities [6]. Furthermore, it is a time-consuming and cumbersome technique, and eggs in stool do not necessarily correlate well with the intensity of exposure to new infections with migrating stages of the parasite that are major contributors to morbidity caused by the parasite [7]. Therefore, it would be useful to have a practical method to measure the intensity of exposure to new infections at the population level. Research on the value of antibody assays for STH infections has been limited to date apart from interesting work on strongyloidiasis [8–10]. One reason for this is that people did not consider antibody testing to be a priority for common infections that can be diagnosed by microscopy. Also, it is commonly believed that antibody tests will not be able to distinguish between current and past infections. Only a handful of studies have evaluated antibody tests for ascariasis. Most of these studies used crude somatic antigen preparations or excretion/secretion (E/S) products derived from larvae or adult parasites cultivated in vitro. These antigens are difficult to procure and generally lack specificity [11–15].

Recently, Vlaminck et al., [16] evaluated a serological test for the detection of ascariasis in fattening pigs based on the detection of IgG antibodies to *A*. *suum* haemoglobin (AsHb) in serum samples by ELISA. The AsHb antigen is highly produced and secreted by both the adult and larval stages [17, 18]. Validation studies with samples from naturally and experimentally infected pigs showed that the antibody assay was superior to detection of eggs in feces for detecting exposure to the infection. A subsequent study showed that antibody reactivity to AsHb correlated with liver pathology caused by migrating *A*. *suum* larvae, and high antibody rates in pig herds were associated with low growth rates (reduced farm productivity) [19].

There are many parallels between ascariasis in pigs and in humans that is caused by the closely related species *A*. *suum* and *A*. *lumbricoides*, respectively. Therefore, the purpose of the present study was to investigate the potential value of anti-AsHb antibody testing for community diagnosis of human ascariasis.

## Materials and Methods

### Parasite cDNA and AsHb

Adult *A*. *suum* parasites were collected with permission from the intestines of infected pigs that were being processed as part of the normal work at a local abattoir in Ghent, Belgium. The fresh worms were snap frozen in liquid nitrogen for subsequent storage at -80°C. Tissue homogenization and mRNA extraction and cDNA construction were essentially performed as described in Rosa et al [20]. The AsHb antigen was purified according to the protocol described by Vlaminck et al [17]. The protein sequence of AsHb was obtained from the Entrez Protein Database of the National Center for Biotechnology Information (NCBI), USA (http://www.ncbi.nlm.nih.gov/protein) with the following accession number: AAA29374.1 (*A*. *suum*). Protein orthologs of AsHb in other nematode species were identified by a BLASTP search on WormBase ParaSite (http://parasite.wormbase.org/) against the available protein sequence databases for the following species (*A*. *suum* (PRJEB80881); *A*. *lumbricoides* (PRJEB4950), *Toxocara canis* (PRJEB533), *Strongyloides stercoralis* (PRJEB528), *Necator americanus* (PRJNA72135), *Ancylostoma ceylanicum* (PRJNA72583), *Enterobius vermicularis* (PRJEB503), *Brugia malayi* (PRJNA10729), *Wuchereria bancrofti (*PRJEB536), *Loa loa* (PRJNA60051), and *Trichuris trichiura* (PRJEB535)). The presence of signal peptides was detected using SignalP 4.1 software [21].

### Cloning of AsHb

The AsHb product was amplified from parasite cDNA by reverse transcriptase- polymerase chain reaction (RT-PCR) using the primer pair AsHbFw (CACCATGCGCTCATTGCTATTATTATCG) and AsHbRv (TCAGTGTTGCTCTTCCTTATGC) and according to the amplification protocol described in Vlaminck et al [17]. The amplified PCR product was cloned into pET100/D-TOPO vector and transformed into One Shot TOP10 competent cells according to the manufacturers protocol (Invitrogen, Carlsbad, CA, USA). Positive colonies were analyzed using PCR and the PCR products were sequenced. One positive transformant was selected and the plasmid was purified using the PureLink HQ Mini Plasmid Purification Kit (Invitrogen). The plasmid DNA was used as template for the amplification of AsHb using the same primers as previously mentioned. The PCR product was sequenced to verify that the insert was in-frame for expression.

### Prokaryotic expression and purification of rAsHb

The AsHb-containing vector construct was transformed into BL21 Star (DE3) One Shot cells and cells were grown in Luria Broth (Miller) (Sigma, St. Louis, MO, USA) containing 50μg/ml carbenicillin (Sigma). Overnight cultures of the transformed bacteria were diluted 1:100 in LB + carbenicillin and grown at 37°C with shaking to an optical density of approximately 0.5–0.8 at 600nm. Protein expression was induced by addition of isopropylthiogalactoside to the culture medium to a final concentration of 1 mM. After 4h of incubation at 37°C under vigorous agitation (250 rpm), *E*. *coli* cells were pelleted by centrifugation and suspended in 1:25 of the initial culture volume of ice-cold RIPA lysis and extraction buffer (G Biosciences, St. Louis, MO, USA), incubated on shaking device at room temperature (RT) for 30 min and then centrifuged for 10 min at 4.500 g. The pellet was suspended again in RIPA lysis and extraction buffer and the previous incubation and centrifugation step was repeated twice more. The final pellet was dissolved in binding buffer (50mM Phosphate buffer, 0.5M NaCl, 10mM imidazole and 7M guanidine hydrochloride, pH 8.0). The recombinant protein was then bound to a HIS-Select Cobalt Affinity Gel column (Sigma) followed by a column wash with 5x column volumes of binding buffer and eluted from the column using elution buffer (50mM sodium phosphate buffer, 500mM NaCl, 7M guanidine hydrochloride, 250mM imidazole, pH 8.0). The eluate was dialyzed overnight against PBS in a Slide-A-Lyzer Dialysis Cassette, 7K MWCO (Thermo Fisher Scientific, Pittsburg, PA, USA) and subsequently concentrated on a Millipore centrifugal filter unit (Millipore, Billerica, MA, USA). Protein dye binding and BCA protein assay (Thermo Fisher Scientific) were used to determine the protein concentration.

### Human plasma samples and assessment of STH infections

Plasma samples were collected from individuals living in Mainang village on Alor Island (Province of East Nusa Tenggara, Timor, Indonesia) as part of a study of the impact of annual MDA with diethylcarbamazine (DEC) combined with albendazole (ALB) on *Brugia timori* and STH infections [22]. Only the most prevalent geohelminths, *A*. *lumbricoides*, hookworm and *T*. *trichiura* were analyzed in this study, since other species such as *Hymenolepis* spp. and *S*. *stercoralis* were only found in a few cases [23]. Baseline prevalence for *A*. *lumbricoides*, *T*. *trichiura* and hookworm in the whole community were 32.2%, 25.3%, and 9.4% respectively [23]. The STH infection rates and egg densities were assessed with single Kato Katz smears in 2002 (prior to any mass drug administration, MDA) and in 2009, two years after the sixth and last annual round of MDA. For these years, community egg output was determined as the sum of EPG of all people divided by the total number of tested individuals. Prevalence rates for STH in stool in 2004 and 2007 were assessed by the formalin ether enrichment method. Additionally, at baseline, 17.6% of the individuals had *B*. *timori* mf in their blood [23].

Plasma samples used in the present study were collected in 2002 prior to the first round of MDA, after 2 rounds of MDA (2004), just prior to the sixth and final round of MDA (2007), and 2 years after the last round of MDA (2009).

Other plasma samples used in this study were from people living in the East Sepik region of Papua New Guinea. Hookworm infection rates in this study area were over 90% and no *A*. *lumbricoides* infections were detected by Kato Katz examination of one stool sample per subject in any of these study communities. Non-endemic control plasma samples were from American subjects in St. Louis, MO, USA.

### SDS-PAGE and Western blot

Protein samples were denatured and reduced in LDS 4x sample buffer (Thermo Fisher scientific) and separated by SDS-PAGE using Bolt 4–12% Bis-Tris Plus minigels (Thermo Fisher Scientific) under reducing conditions and either stained with SimplyBlue Safestain Coomassie stain (Invitrogen) for the visualization of the proteins or transferred onto nitrocellulose membranes for immunostaining as previously described [24]. After blotting, nitrocellulose membranes were blocked at room temperature for 1hr with 5% non-fat dry milk (BioRad, Hercules, CA, USA) in PBS, and then incubated with human plasma diluted 1:50 in PBS + Tween20 (0.05%) (PBST) and incubated at room temperature (RT) for 2h. After incubation with a secondary antibody (mouse anti human IgG4 pFc-HRP (Southern biotech, Birmingham, AL, USA), blots were washed with PBST and antibody binding was detected using CN/DAB Substrate kit (Thermo Fisher Scientific).

### Deglycosylation of AsHb and analysis of the carbohydrate groups

AsHb was deglycosylated with PNGase F according to the manufacturer’s protocol (New England Biolabs, Ipswich, MA, USA). Briefly, 10μg of AsHb was mixed with 10x denature buffer and H_2_O to make a 20μl total reaction volume that was denatured at 100°C for 10 min. This was followed by addition of 10X Glycobuffer 2, 10% NP-40 and 1μl of PNGase F (500,000 U/ml) and H_2_O to obtain a total reaction volume of 40μl that was incubated at 37°C for 2h. Finally, the deglycosylated product (dAsHb) was passed over a detergent binding spin column (Thermo Fisher Scientific) to remove the detergent that was added during the deglycosylation reaction. RNAseB treated with PNGase F was included as deglycosylation control.

For mass spectrometric (MS) analysis, N-glycans released with PNGase-F from 5μg of AsHb were labeled with 2-aminobenzoic acid (anthranilic acid, AA), as described [25]. MALDI-TOF-MS was performed in the negative ion reflector mode on an Ultraflextreme instrument (Bruker Daltonics, Germany) using DHB as matrix, as described [26]. Putative glycan structures were assigned on the basis monosaccharide compositions deduced from the observed *m/z* values.

Phosphorylcholine (PC) specific monoclonal antibodies (TEPC-15) (Sigma) were used to detect the presence of PC in AsHb and dAsHb by Western blot and ELISA. Anti-PC antibodies were detected with HRP conjugated anti-mouse IgA (Sigma) and nitro-blue tetrazolium/5-bromo-4-chloro-3′-indolyphosphate substrate (Sigma). PC linked to bovine serum albumin was used as a positive control.

### Detection of IgG4 antibodies to AsHb by ELISA

Previous serological experiments performed by Santra et al., 2001 [13] and Chatterjee et al., 1996 [14] showed that human IgG4 responses to a fractionated adult E/S antigen of *Ascaris* were superior in reactivity and also showed less cross reactivity than IgG1, IgG2 and IgG3 subclass antibodies in sera from patients infected with hookworm, *Trichuris* and *Strongyloides*. This, in combination with the results from earlier experiments performed in the lab, led to the decision to use the IgG4 subclass antibody as detecting antibody in the immunological assays described in this study.

Antigen (AsHb, rAsHb or dAsHb) was coated at a concentration of 1μg/ml overnight at 4°C on Nunc Maxisorp flat-bottomed 96 well plates (Sigma) in 100μl coating buffer (0.05M carbonate/bicarbonate buffer pH9.6). Following incubation, plates were washed 3 times with wash buffer (PBST: 0.05% PBS-tween 20, pH 7.2). Nonspecific binding sites were blocked by dispensing 100 μl of PBS with 5% FCS in each well and incubating the plates for 2h at 4°C. For the inhibition ELISA, an extra blocking step was included where PC-groups on the AsHb were blocked by incubating the coated wells with TEPC-15 antibodies diluted 1:500 in blocking buffer for 2 h. After blocking, plates were washed as before and plasma or antibody samples were added to the wells. Plasma samples were diluted 1:50 in PBST and 100 μl of each sample was tested in duplicate. Plates were incubated for 2h at RT and afterwards washed as previously described. Secondary antibodies (mouse anti-human IgG4 pFc’-HRP (Southern biotech)) were diluted 1:2,000 in blocking buffer and plates incubated for 1h at RT. Finally, plates were washed and 100μl of the o-phenylenediamine dihydrochloride substrate solution (Thermo Fisher Scientific) was added to each well. The substrate reaction was stopped after 10 minutes by adding 50μl of stop solution (4M H_2_SO_4_) and optical densities at 490nm were recorded. The cutoff for positivity was calculated as the arithmetic mean OD + 3 times the standard deviation obtained with 10 non-endemic plasma samples from St. Louis, Missouri, USA.

### Statistical analysis

All statistical analyses were performed using GraphPad Prism v6.0 software (La Jolla, CA, USA). Infection prevalence rates at baseline and subsequent time points were compared with the McNemar test. Infection intensities (EPG) and ELISA OD values for paired samples were compared using the Wilcoxon signed rank test. Correlations between EPG and AsHb ELISA OD values were assessed with the Spearman’s rank correlation test. The statistical significance of differences in ELISA OD values obtained with different antigens (AsHb, dAsHb and rAsHb) was assessed with the Wilcoxon signed rank test.

### Ethical clearance

The Ethics Committee of the University of Indonesia, Jakarta approved the sample collection in the Alor Island study as previously described [22]. The Institutional Review Boards at Case Western Reserve University and the Papua New Guinea Medical Research Advisory Committee approved the protocol for sample collection, and all study participants provided informed consent. The Institutional Review Board at Washington University School of Medicine waived the need for an additional review for our use of de-identified human serum samples for this *in vitro* study.

### Accession numbers

Genbank: AAA29374.1. Wormbase: GS_08371, ALUE_0001899801, ALUE_0001446901, NECAME_07759 ANCCEY_14143, TCNE_0001552801.

## Results

### Detection of AsHb by human antibodies on Western blot

**Fig 1:**
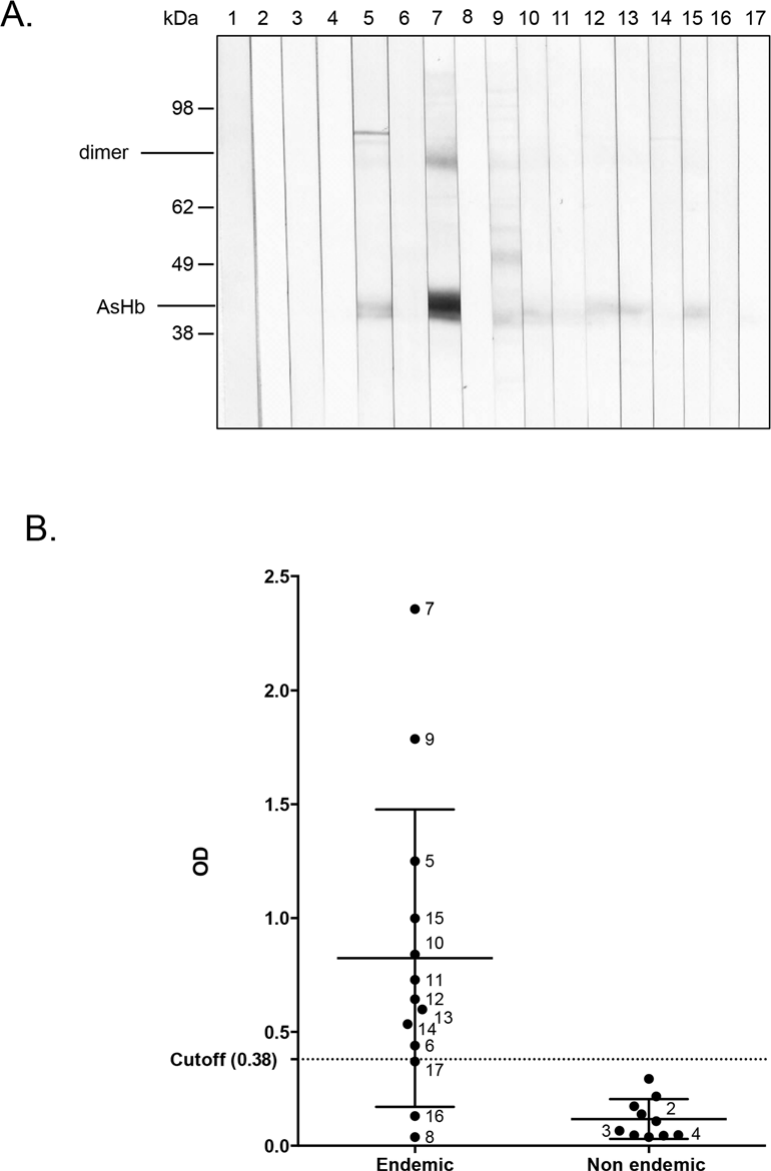
Native AsHb is recognized by human IgG4 antibodies in plasma samples from individuals living in an *A*. *lumbricoides* endemic area. (A) The recognition of AsHb by human IgG4 antibodies on Western blot; lane 1 = conjugate control, lane 2–4 = Non-endemic controls, lane 5–10 = endemic plasma samples without visible parasite eggs in the stool, lane 11–17 = endemic plasma samples from individuals with *A*. *lumbricoides* eggs in the stool. (B) ELISA OD values for the same endemic plasma samples and 10 non-endemic plasma samples. The number next to each point on the dot plot corresponds with the lane numbers for the Western blot. The OD values of the 10 non-endemic individuals were used to calculate the cutoff (0.380) indicated on this plot by a dotted line.

In order to evaluate whether antibodies of infected humans were detecting AsHb, a select number of endemic and non-endemic control plasma samples were first used to evaluate the recognition of AsHb by Western blot and ELISA. Six of 8 endemic plasma samples with proven *A*. *lumbricoides* infection (>25 EPG) and 4 of 5 individuals with negative stool examinations had IgG4 antibodies that recognized AsHb (**Fig 1)**. In contrast, none of the non-endemic control plasma samples detected AsHb on Western blot or showed strong reactivity on ELISA. A cut-off for ELISA positivity was determined, based on the OD values of the 10 non-endemic plasma samples that were tested. The cut-off was set at an OD of 0.38.

Specificity of the AsHb IgG4 Western blot was also assessed with plasma samples collected in an area in Papua New Guinea where hookworm infection is nearly universal but ascariasis and trichuriasis are absent. Since 7 of the 12 samples tested had IgG4 antibodies to AsHb **(S1 Fig)**, it appears that some people with hookworm infections develop IgG4 antibodies to AsHb to some degree. A BLASTP search using the AsHb protein sequence obtained from GenBank (AAA29374.1) on Wormbase Parasite revealed two sequences in *A*. *lumbricoides* (ALUE_0001899801 and ALUE_0001446901) with >99% amino acid sequence identity. Both sequences form a perfect alignment with AsHb (**S2 Fig**). Both *N*. *americanus* and *A*. *ceylanicum* have orthologs to AsHb (NECAME_07759 and ANCCEY_14143 respectively) with sequences that were shorter than the AsHb (88 AA and 97 AA respectively) with sequence identity in the overlapping region of 51.1% and 46.4% and E values of 1e^-25^ and 2.7e^-24^ respectively. The ortholog in the canine ascarid *Toxocara canis* (TCNE_0001552801) has 69.6% amino acid sequence identity and also showed to contain a signal peptide. No AsHb orhtolog (E-value < 1e^-05^) was present in *Trichuris spp*. (**S1 Table**).

### Changes in STH infection rates and antibody prevalence rates to AsHb following mass drug administration in Mainang village, Indonesia

This part of the study used ELISA to detect human IgG4 antibodies to AsHb in plasma samples collected before and after MDA. All parasitological and serological data obtained in this study is provided as supplementary information (**S2 Table**). STH prevalence results before and after MDA are shown in **Table 1**. Although the prevalence of *A*. *lumbricoides* infection decreased significantly from 38.2% to 15.7% after 2 years of MDA (a 58.9% reduction; P < 0.01), the prevalence rate rebounded to 24.4% in 2007 (a 36.1% reduction from baseline; P < 0.01) and to 29.5% in 2009 (a 22.8% reduction from baseline, P = 0.09). In contrast, the average egg output in the community was 81.6% lower in 2009 than in 2002 (128.2 vs. 697.9, P < 0.01). Hookworm and *T*. *trichiura* infections were also reduced by MDA (both 1.8% in 2007), however in 2009 hookworm infection rates returned to pre-MDA levels (10.4%) and *T*. *trichiura* rates also rebounded (2.3%) [22]. The average community egg outputs for hookworm and whipworm did not change significantly between 2002 and 2009 (5.4 to 6.9 and 3.1 to 1.4 respectively).

**Table 1:** Prevalence of *A*. *lumbricoides*, hookworm and *T*. *trichiura* infections based on stool examination and serological examination for *A*. *lumbricoides* infections during and after mass drug administration with DEC + albendazole in a subset of the treated population that was studied from 2002 to 2009 on Alor Island, Indonesia.

	Year of the study				
	2002	2004	2007	2009	% reduction 02 vs 09
**No of stool samples examined (qualitatively/quantitatively)**	280/280	280/0	279/0	173/173	
***A*. *lumbricoides* % positive (n)**	38.2 (107)	15.7 (44)	24.4 (68)	29.5 (51)	22.9
***Ascaris* community egg output^1^**	697.9	/	/	128.2	81.6*
**Hookworm % positive (n)**	8.9 (25)	3.6 (10)	1.8 (5)	10.4 (18)	-16.5
**Hookworm community egg output^1^**	5.4	/	/	6.9	-27.8
***T*. *trichiura* % positive (n)**	6.8 (19)	0.4 (1)	1.8 (5)	2.3 (4)	65.9
***T*. *trichiura* community egg output^1^**	3.1	/	/	1.4	53.0
**No of plasma samples**	476	399	100	91	
**% seropositive samples (n)**	67.6 (322)	22.6 (90)	23.0 (23)	14.3 (13)	78.9*
**No of samples with both EPG and serology data for *Ascaris***	240	220	68	64	
**% that are sero + and egg + (n)**	27.5 (66)	4.5 (10)	13.2 (9)	4.7 (3)	
**% that are sero + and egg–(n)**	37.9 (91)	15.0 (33)	14.7 (10)	6.3 (4)	
**% that are sero–and egg + (n)**	10.0 (24)	10.5 (23)	14.7 (10)	26.6 (17)	
**% that are sero–and egg–(n)**	24.6 (59)	70.0 (154)	57.4 (39)	62.5 (40)	

(* P < 0.01)

^**1**^ Community egg output = sum of EPG / total number of tested persons.

**Fig 2:**
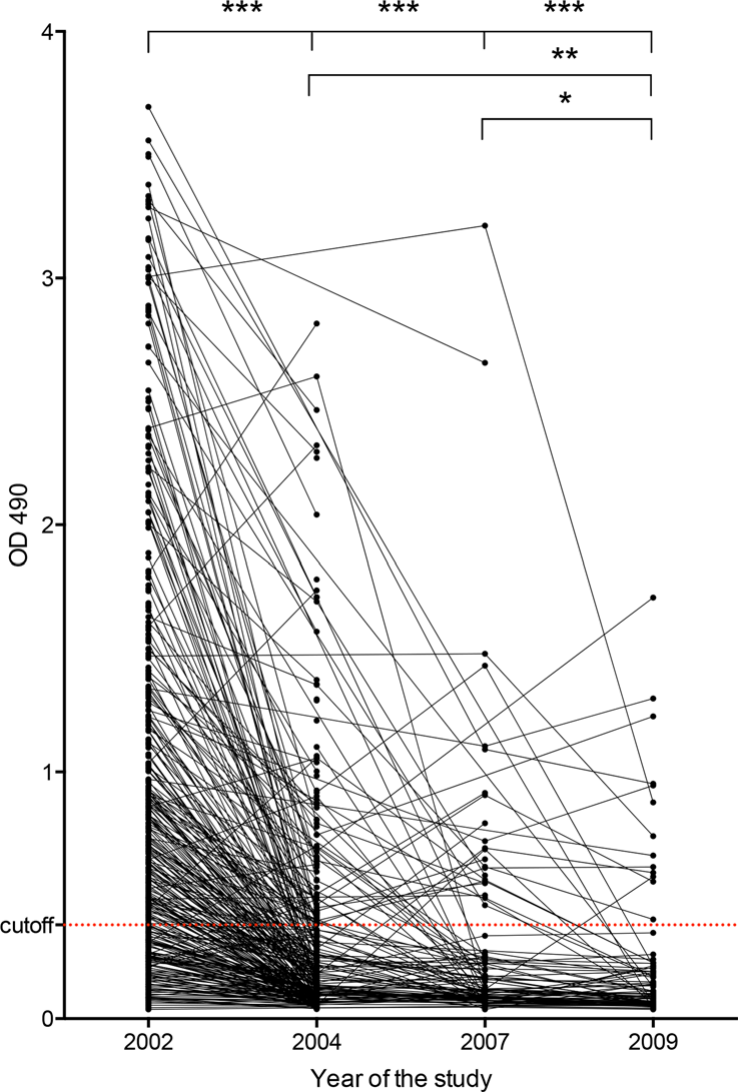
Human IgG4 response against native AsHb is reduced by MDA intervention. Plasma samples were analyzed at baseline (2002), after two years of MDA (2004), at the end of the trial, prior to the final MDA (2007), and 2 years after the end of the trial (2009). Antibody reactivity decreased significantly after MDA. The lines connect plasma samples from the same individuals collected in different years of the study. The red dotted line represents the OD cutoff of 0.380 (* P < 0.05, ** P < 0.01, *** P < 0.001).

A total of 1,066 plasma samples were tested by AsHb ELISA, and these included samples collected prior to any MDA and at intervals following multiple rounds of MDA (**Table 1**). Employing the earlier determined OD cut-off for the ELISA, 67.6% of the individuals were seropositive at baseline (**Table 1, Fig 2 and S3 Fig**). After two years of MDA, seroprevalence was significantly reduced to 22.6% in 2004 (P < 0.001) and this was unchanged in 2007 (23.0%). The seropositivity rate in 2009 had further decreased in comparison to 2007 (14.3%, P < 0.05).

In both 2002 and 2009 there was no significant relationship between AsHb ELISA OD values and stool egg counts for any of the STH (**S4 Fig**). In 2002, 66 of 90 egg positive (73.3%) and 91 of 150 (60.7%) egg negative individuals were seropositive by anti-AsHb ELISA. However, in 2009 only 3 of 20 egg positive (15%) and 4 of 44 (9.1%) egg negative individuals were seropositive.

**Fig 3:**
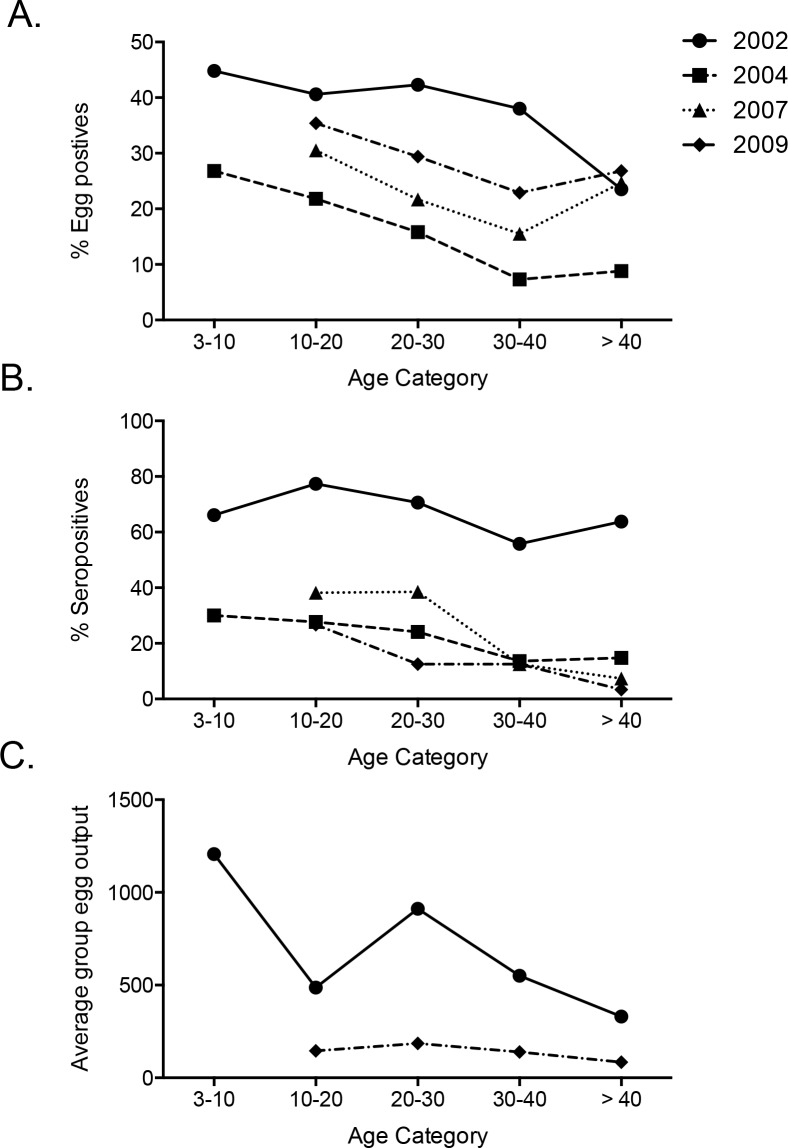
Changes in age-specific *Ascaris* infection rates, AsHb antibody rates, and average egg output per age group for different times before and after MDA. Prevalence based on eggs in the stool (A) or serology (B) shows a notable reduction over all age categories between the year 2002 and 2004. While the prevalence goes back up in all age categories based on the presence of eggs in the stool in subsequent years, the percentage seropositive individuals remains low. (C) The average group egg output (sum of EPG divided by the number of people analyzed per group) is also reduced between 2002 and 2009 over all age categories. Too few subjects (less than 5) younger than 10 years of age were tested in 2007 and 2009 to produce meaningful data for this age category.

There was a drastic and significant reduction in both egg prevalence and seroprevalence between 2002 and 2004 in all age groups. Egg prevalence increased again in 2007 and 2009 across all age groups until it almost reached pre-treatment levels whereas seroprevalence did not change after 2004. Similarly, the mean number of eggs excreted by the people in a specific age category was also significantly reduced from 2002 to 2009 over all age categories (**Fig 3**).

### Recognition of AsHb, dAsHb and rAsHb by human IgG4 antibodies

**Fig 4:**
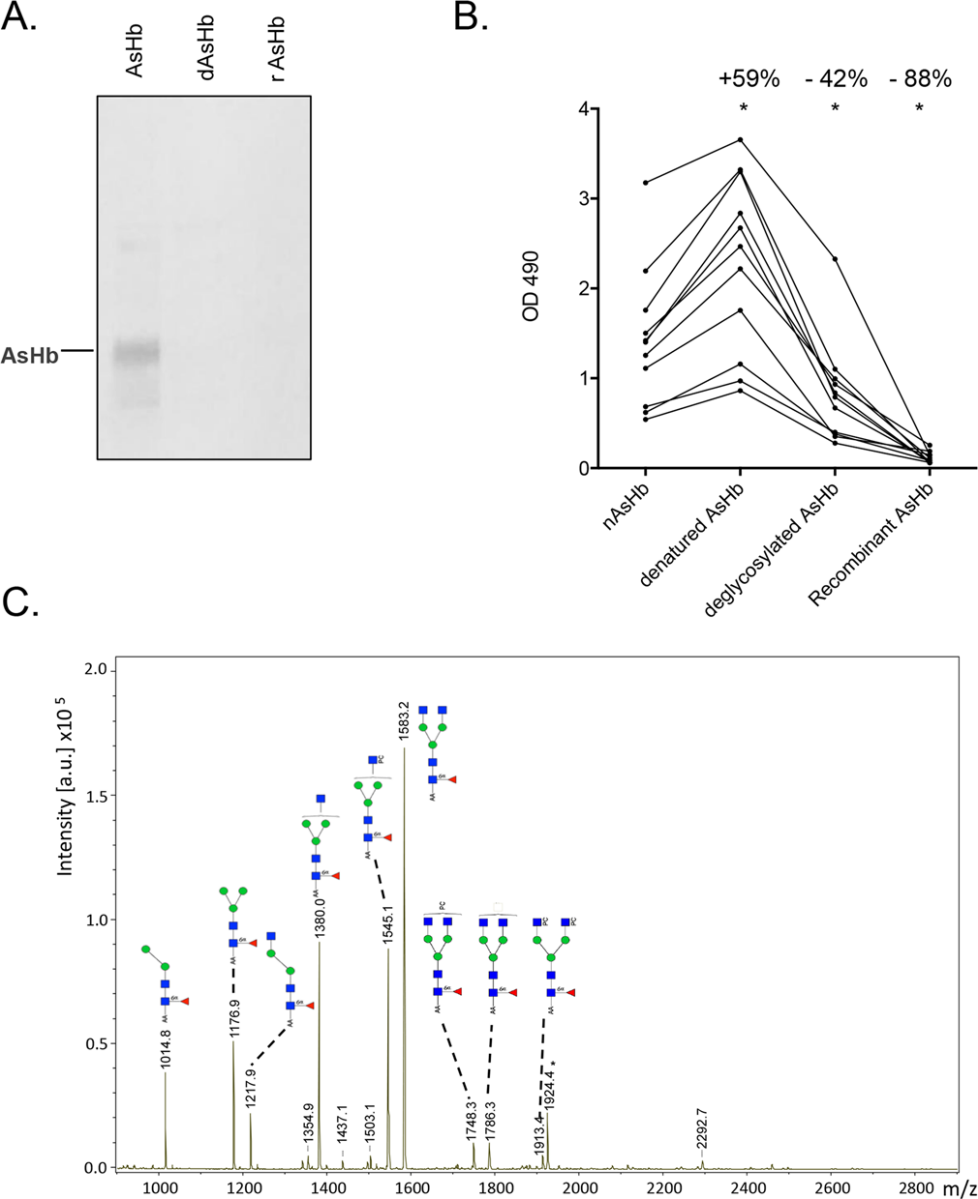
Characterization and importance of N-glycans for the recognition of AsHb by human IgG4 antibodies. (A) Recognition of AsHb, PNGase F deglycosylated native AsHb (dAsHb), and recombinant AsHb produced in *E*. *coli* (rAsHb) on Western blot by IgG4 of pooled *A*. *lumbricoides* infected human plasma samples. (B) Comparison of ELISA results obtained with IgG4 antibodies from 10 humans with ascariasis *vs*. AsHb, denatured AsHb (den AsHb), dAsHb and rAsHb. The average % change relative to AsHb is shown above the bars with * for differences with P values of < 0.01. (C) Anthranilic acid (AA)-labeled glycans released by PNGase-F from AsHb identified by MALDI-TOF-MS. The major glycans detected were core-α1,6-fucosylated trimannosyl N-glycans with GlcNAc residues that were partially substituted by phosphorylcholine (PC). Red triangle, fucose; blue square, *N*-acetylglucosamine; green circle, mannose; white square, *N*-acetylhexosamine; *, unidentified non-glycan signal.

In order to work towards better standardization of the test, we also evaluated whether infected human sera would recognize recombinant AsHb. The AsHb gene was cloned from adult worm cDNA and expressed in *E*. *coli*. The protein profile of the purified rAsHb was identical to that of AsHb after Coomassie staining (**S5 Fig**). Antibodies in a pooled plasma sample from humans with *Ascaris* infection did not bind to rAsHb by Western blot (**Fig 4**). To test whether the presence of N-glycan groups on AsHb was important for immune recognition, the native AsHb was deglycosylated with PNGase F to remove any N-linked glycans. A shift in molecular weight was seen in the PNGase F deglycosylated AsHb (dAsHb), indicating the removal of N-linked glycans. This molecular weight shift was not visible after treatment of the rAsHb with PNGase F indicating the absence of PNGase F digestible carbohydrate groups (**S5 Fig**). Antibodies in pooled plasma from *A*. *lumbricoides* infected individuals did not bind to dAsHb by Western blot.

In order to quantify this effect, IgG4 reactivity of 10 plasma samples from Indonesian individuals from 2002 to AsHb, denatured AsHb, dAsHb and rAsHb were evaluated by ELISA (**Fig 4**). After normalization of the data using the AsHb OD 490 as reference, a significant relative increase in antibody binding (59%, P < 0.05) was seen after denaturing the AsHb. The opposite was true for dAsHb and for rAsHb that were less immunoreactive than the native antigen (-42%, P < 0.05 and -88%, P < 0.01, respectively.

### Identification of PNGase F released glycans of AsHb

In order to further characterize the glycans present on AsHb that were important for immune-recognition of the antigen, the N-glycan structures were removed from the protein backbone by PNGase F and analyzed by MALDI-TOF-MS. The glycan spectrum of the released N-glycans of AsHb is shown in **Fig 4**. Major [M-H]^-^ signals were observed at *m/z* 1176.9, 1380.0 and 1583.2, derived from glycans with the compositions F_1_N_2_H_3_, F_1_N_3_H_3_ and F_1_N_4_H_3_ (F, fucose; *N*-acetylhexosamine, H, hexose) glycans, respectively, interpreted as α1,6-fucosylated trimannosyl N-glycan core structures substituted with 0–2 GlcNAc residues (indicated in **Fig 4**). An additional major signal was observed at m/z 1545.1 (F_1_N_3_H_3_+165.1) indicative for the phosphorylcholine (PC) substituted variant of F_1_N_3_H_3_ which is in line with previous observations for N-glycans of *A*. *suum* [27]. Further, minor signals at m/z 1748.3 (F_1_N_4_H_3_PC), 1786.3 (F_1_N_5_H_3_) and 1913.4 (F_1_N_4_H_3_PC_2_) were observed indicating that additional substitutions with HexNAc residues and PC can occur. Incubation of both AsHb and dAsHb with mouse anti-PC monoclonal antibodies (TEPC-15) proved the presence of PC in native AsHb and its absence after deglycosylation with PNGase F (**S6 Fig**). The recognition of AsHb by human IgG4 antibodies was not significantly reduced when AsHb was pre-incubated with anti-PC antibodies before addition of positive human plasma samples (**S6 Fig**).

## Discussion

The results of this study suggest that measurement of antibodies to AsHb may be a useful approach for assessing exposure of human populations to *A*. *lumbricoides* infection. Egg excretion per person and antibody rates decreased in parallel following MDA while the infection prevalence rate in 2009 was not very different from the baseline rate. The implementation of MDA probably reduced the number of *Ascaris* eggs that were excreted in the environment. As a result, the ingestion of infective eggs and exposure to migrating stages of the parasite is likely to have decreased in all age categories in the population. This may explain why the antibody rate decreased further between 2007 and 2009 despite suspension of the MDA program after 2007. However, since we do not have quantitative coprological data for the years 2004 and 2007, we do not have precise information on the impact of MDA on infection intensities between 2002 and 2007.

Despite the limited sequence similarity between the AsHb orthologs identified in both hookworm species, our results confirm antigenic cross reactivity between hookworm and *A*. *lumbricoides* [28]. Thus, reduced antibody reactivity to AsHb after MDA may have been partly due to the effects of MDA on hookworm prevalence or transmission. However, the significant rebound in hookworm prevalence that occurred between 2007 and 2009 (from 1.8% to 10.4%) was not associated with a rise in antibody rates to AsHb. This study did not investigate antibody responses to AsHb in people infected with *T*. *trichiura*. However, the absence of an AsHb homologue in the *Trichuris* genome and the fact that antibodies in sera from pigs experimentally infected with *T*. *suis* had little if any reactivity with AsHb [16] suggest that humans infected with *T*. *trichiura* who have not been infected with *Ascaris* are not likely to have significant antibody responses to AsHb.

We acknowledge that using the AsHb ELISA would be too insensitive to identify or diagnose active *A*. *lumbricoides* infection in an individual. However, it is important to notice that the goal of our study was to evaluate the use of this serological test as diagnostic marker for exposure on a community level and not for individual diagnosis of infection. Unlike for *A*. *suum* infections in pigs, where experimentally infected pigs can serve as “true gold standard”, there is no such standard available to assess the sensitivity of a serological tool for diagnosis of STH infections in humans [6]. However, since this ELISA would be used to assess transmission intensity on a community level, this is not a major obstacle.

Recombinant antigens are often preferred for serodiagnosis of parasitic infections. One reason for this is that native antigens are sometimes difficult to obtain and purify. However, in the case of AsHb, the antigen is relatively easy to purify from *A*. *suum*. In addition, antibodies from infected individuals were only weakly reactive with recombinant AsHb produced in this study or with AsHb after treatment with PNGase F. This finding suggests that IgG4 human antibodies to AsHb are mostly directed against N linked glycan epitopes present on the native antigen. Shared carbohydrate epitopes pose a challenge for developing specific serology tests for helminth infections. Parasites with AsHb orthologs with low amino acid sequence identity may contain the same or similar carbohydrate epitopes that could be responsible for antigenic crossreactivity.

Mass spectrometric analysis of the glycans released by PNGase F treatment of AsHb detected PC-substituted GlcNAc moieties. Blocking the PC-group with TEPC-15 antibodies did not result in a significant blocking of binding of human antibodies to AsHb by ELISA. This result suggests that other glycans motifs may be important antigenic components of AsHb and it is consistent with a previous study that reported that humans do not develop IgG4 subclass antibodies to the PC epitope [29].

The simplicity, affordability and speed of the Kato Katz test has made it the most widely used method for estimating STH infection rates and intensities in large scale control programs [30]. This has important policy implications, because current WHO guidelines for STH preventive chemotherapy are based on infection prevalence rates as assessed by a single Kato-Katz smear with little attention paid to intensity [31]. However, prevalence is not everything. Because of the high degree of aggregation of STH infections, significant reductions in average worm loads may result in small or unnoticeable changes in prevalence rates [32]. Also, infection intensities for ascariasis are often reported according to the broad WHO categories of light (< 5,000 EPG), moderate (5,000–50,000 EPG) or heavy (>50,000 EPG). However, it is unclear whether these categories are appropriate for use in all endemic regions, because of considerable geographic variability in egg production per adult female *Ascaris* worm [33].

The use of diagnostic tools that are based on the detection of *Ascaris* eggs or DNA in the stool has significant shortcomings when it comes to the accurate estimation of true prevalence or intensity of the infection. For one thing, only a small fraction of the total number of parasite larvae that migrate through the body ever develop into adult worms in the intestine. For worms that reach the intestine, egg output per worm can vary widely because of different parasite sex ratios, the age distribution of the adult worms, and host immunity [7]. Thus the absence of *A*. *lumbricoides* eggs in the stool does not necessarily prove there has been no recent infection or exposure with larval stages. Animal studies have shown that larval stages contribute significantly to morbidity caused by *Ascaris* infections [34, 35], and this should be considered as part of the health impact of ascariasis in humans as well.

Our results show that IgG4 antibody responses to AsHb do not correlate with *A*. *lumbricoides* egg output. This is consistent with results from other studies that have compared anti-*Ascaris* antibody responses to adult worm counts or EPG in stool [12, 36]. Furthermore, several studies have shown that anti-*Ascaris* antibody responses are rather dependent on exposure and infection intensity as opposed to being protective or predictive of future levels of infection [36–39].

A significant percentage of people in Alor Island who had *Ascaris* eggs in their stool lacked IgG4 antibodies to AsHb, and this situation became more common later in the trial when community egg loads were reduced. It is interesting that a number people with more than 500 EPG were seronegative in 2009. While there are several possible explanations for this, we favor the idea that antibody responses to AsHb are stimulated much more by new infections when larvae are migrating through tissues and than by the presence of low numbers of adult worms in the intestinal lumen. When the infection pressure has been reduced to low levels, exposure to new infections with migrating larvae may not be sufficient to induce or maintain IgG4 antibodies to AsHb. Furthermore, experimental infections in pigs have already shown that the number of adult worms in the gut seems almost inversely correlated with the number of eggs ingested [40, 41]. Hence, with lower infection intensities, the chance of larvae establishing in the gut and developing into adult worms increases. It would be interesting to elucidate the effect of infection dose on anti-AsHb responses in pigs, and this might help to explain changes in antibody rates in human populations when infection pressure declines following MDA.

Although AsHb serology is not sensitive for detecting active *Ascaris* infections in individuals, it appears to be a promising new tool for quantifying exposure to *Ascaris* infection at the community level. That is to say, it may provide a useful measure of egg input and incoming infection in communities even though it is not sensitive for predicting the presence of eggs in the stool in individuals. Serological surveys for antibodies to AsHb and other STH antigens could be an attractive alternative to stool examination for integrated post-MDA surveillance programs for lymphatic filariasis (LF) and STH, because post-MDA surveys for LF collect finger prick blood samples for use in point of care serology tests [3], and finger prick blood could also be used for STH serology. AsHb serology could also be used as an alternative to Kato-Katz test for mapping the distribution of STH infections, because it should be useful for identifying areas with high transmission rates that have the highest need for intervention.

The crossreactivity with hookworm and possibly *Strongyloides stercoralis* and *Toxocara* spp. limits the value of AsHb serology if one is interested in ascariasis alone. Work to develop a more species-specific antibody assay is ongoing. However, hookworm and *Ascaris* crossreactivity may not be a major flaw for the practical use of AsHb serology, since the same drugs are used to treat both of these infections. We believe that this study has provided a useful proof of principle for the value of antibody serology as an epidemiological tool for assessing STH transmission pressure in populations and for monitoring the impact of STH intervention on transmission pressure. We have shown that AsHb antibody rates correlate well with egg output per person in populations, and they are also likely to correlate well with recent egg *input* in individuals. However, additional research is needed on this topic. First, it will be necessary to confirm the findings from this study with samples from other areas with high rates of ascariasis. Second, it would also be interesting to evaluate changes in serology over time in different subpopulations or to use serology to try and pinpoint when children are first exposed to STH.

## Supporting Information

S1 Table Homologs of AsHb determined by NCBI BLAST search of the nr database and detection of possible secretory signal peptide. (XLSX)

S2 Table Raw data file with all parasitological and serological data used in this study. (XLSX)

S1 Fig Some humans living in a hookworm endemic area develop IgG4 antibodies to AsHb. Lane 1 = conjugate control, lane 2 = positive control pooled plasma from *A*. *lumbricoides* infected individuals, lane 3 = pooled non-endemic control plasma, lane 4–15: plasma from individuals from a hookworm endemic area in Papua New Guinea.
(TIF)

S2 Fig Alignment report showing protein alignment of AsHb (AAA29374.1 and GS_08371) and two sequences (ALUE_0001446901 and ALUE_0001899801) identified in *A*. *lumbricoides* (AA that differ from consensus are indicated in red). (TIF)

S3 Fig Human IgG4 response against native AsHb of a subset of 42 individuals that were tested at each time-point in the study. Plasma samples were analyzed at baseline (2002), after two years of MDA (2004), at the end of the trial, prior to the final MDA (2007), and 2 years after the end of the trial (2009). The lines connect plasma samples from the same individuals collected in different years of the study. The red dotted line represents the OD cutoff of 0.380 (* P < 0.05, ** P < 0.01, *** P < 0.001).
(TIF)

S4 Fig Correlation plots for IgG4 antibody levels against AsHb and EPG levels for *A*. *lumbricoides* (A), hookworm (B) and *T*. *trichiura* (C) at baseline (2002) and 2 years after 6 rounds of MDA (2009).
(TIF)

S5 Fig Amplification of the AsHb gene product, purification and deglycosylation of AsHb and rAsHb. **(A)** AsHb cDNA was amplified from total adult *A*. *suum* cDNA. (B) The AsHb was cloned and expressed in *E*. *coli* and purified by affinity chromatography. The native (AsHb) and recombinant AsHb (rAsHb) look identical on Coomassie stained SDS-PAGE gel. (C) A Coomassie stained SDS-PAGE gel of the AsHb and rAsHb before and after deglycosylation with PNGase F.
(TIF)

S6 Fig Presence of Phosphorylcholine (PC) on AsHb. (A) The recognition on Western blot of AsHb, dAsHb and PC linked to bovine serum albumin (BSA-PC) by anti-PC monoclonal antibodies (TEPC-15). (B) A Tukey box plot representing the values of 20 *A*. *lumbricoides* positive (EPG) plasma samples shows no significant (NS) difference in the intensity of detection of AsHb and AsHb that was blocked with TEPC-15 antibodies (1:500 dilution) for 2 hours.
(TIF)
