# Supplementary figures and images for: Community Rates of IgG4 Antibodies to Ascaris Haemoglobin Reflect Changes in Community Egg Loads Following Mass Drug Administration
Source: PLoS Negl Trop Dis. 2016 Mar 18;10(3):e0004532. doi: 10.1371/journal.pntd.0004532 (PMC4798312; doi:10.1371/journal.pntd.0004532)

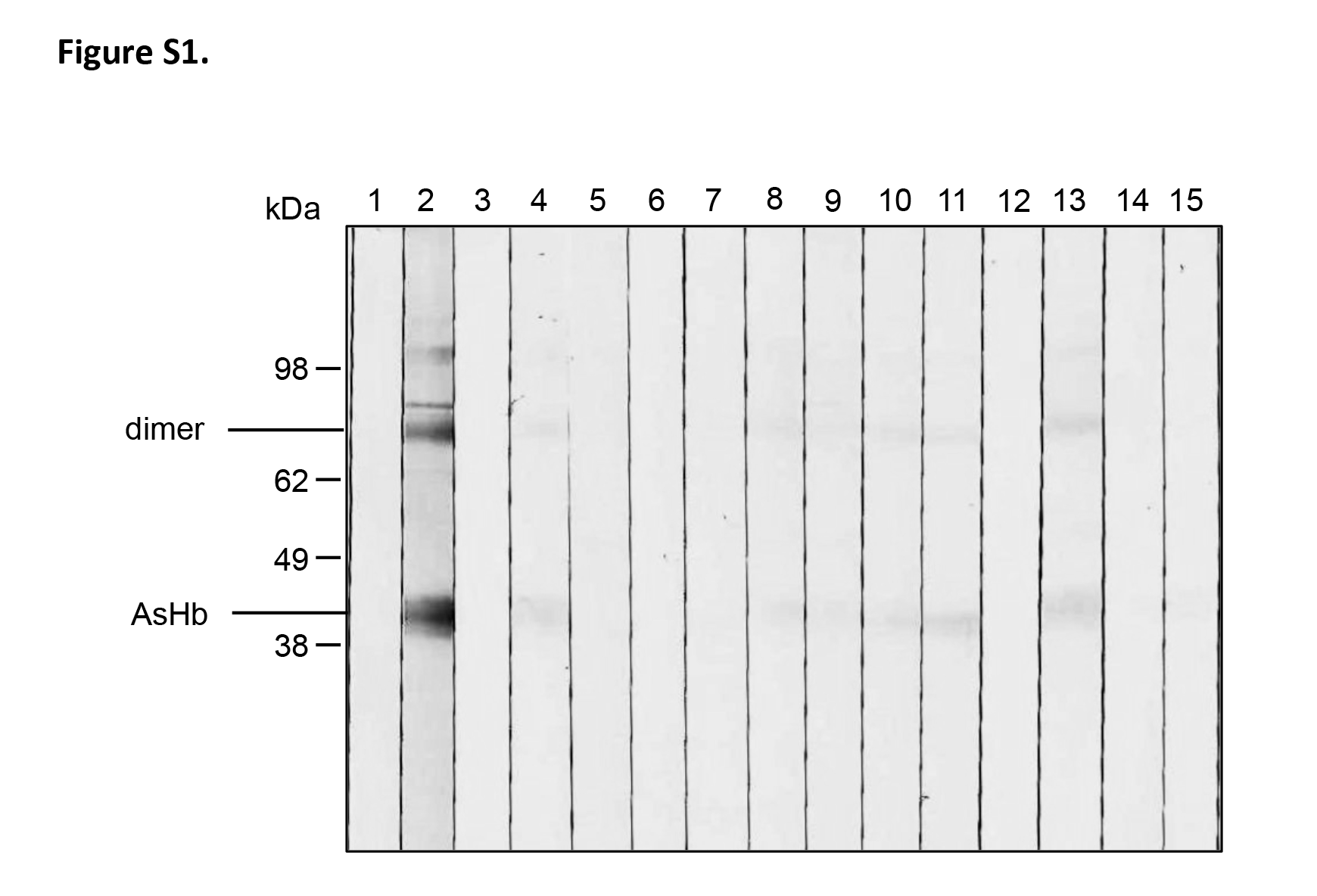

Supplement: S1 Fig — Lane 1 = conjugate control, lane 2 = positive control pooled plasma from A. lumbricoides infected individuals, lane 3 = pooled non-endemic control plasma, lane 4–15: plasma from individuals from a hookworm endemic area in Papua New Guinea. (TIF) [file pntd.0004532.s003.tif]

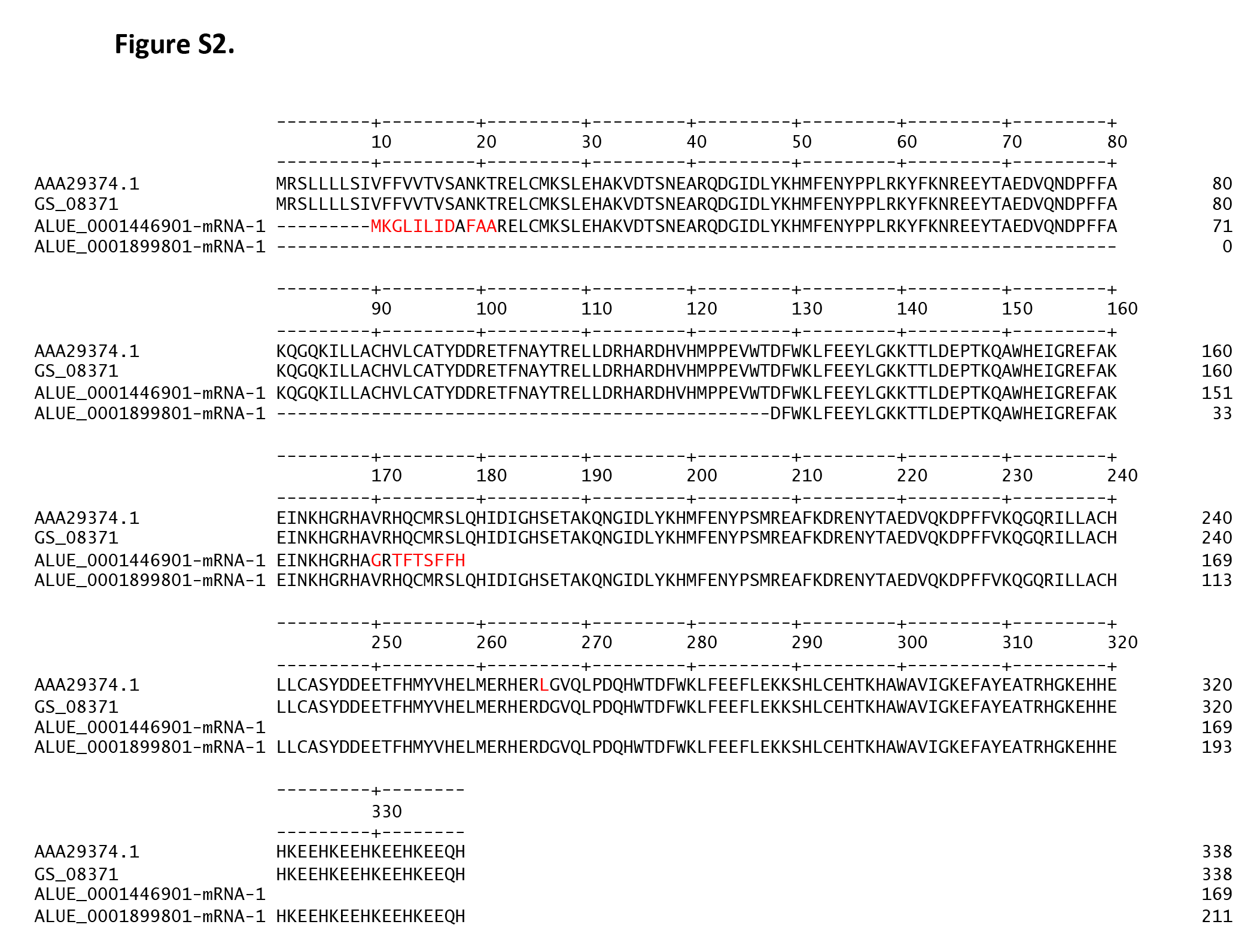

Supplement: S2 Fig — (TIF) [file pntd.0004532.s004.tif]

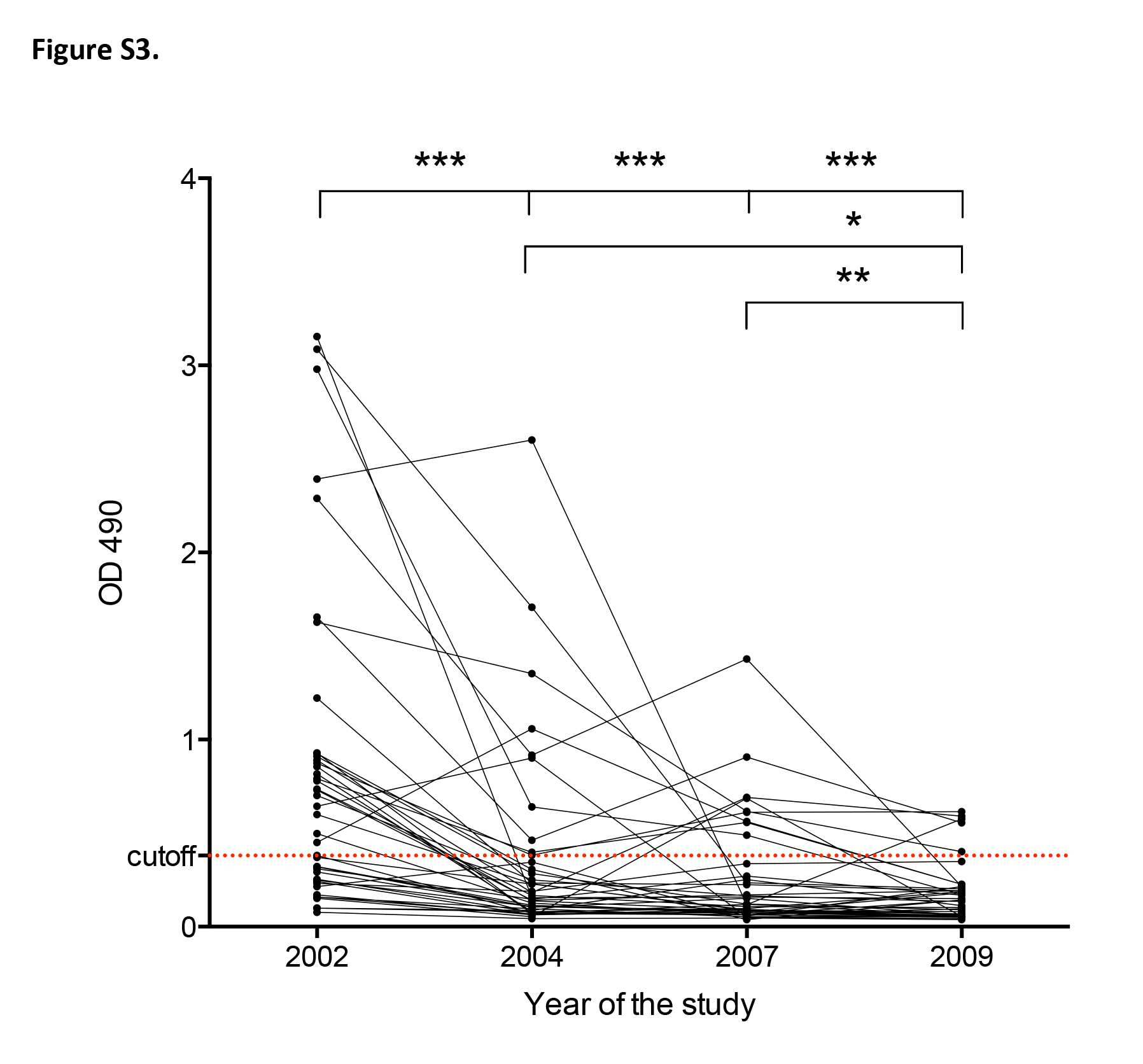

Supplement: S3 Fig — Plasma samples were analyzed at baseline (2002), after two years of MDA (2004), at the end of the trial, prior to the final MDA (2007), and 2 years after the end of the trial (2009). The lines connect plasma samples from the same individuals collected in different years of the study. The red dotted line represents the OD cutoff of 0.380 (* P < 0.05, ** P < 0.01, *** P < 0.001). (TIF) [file pntd.0004532.s005.tif]

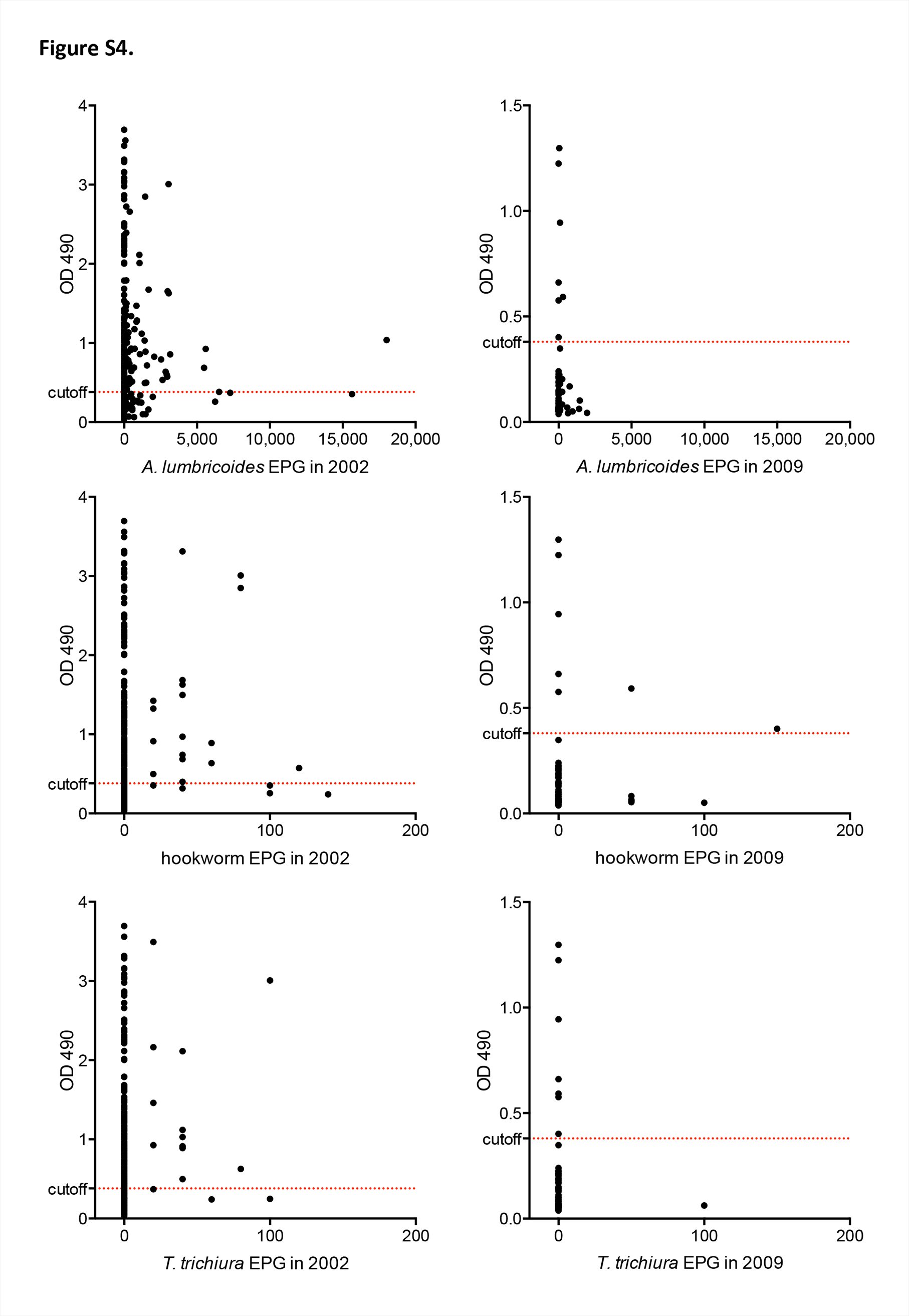

Supplement: S4 Fig — Correlation plots for IgG4 antibody levels against AsHb and EPG levels for A. lumbricoides (A), hookworm (B) and T. trichiura (C) at baseline (2002) and 2 years after 6 rounds of MDA (2009). (TIF) [file pntd.0004532.s006.tif]

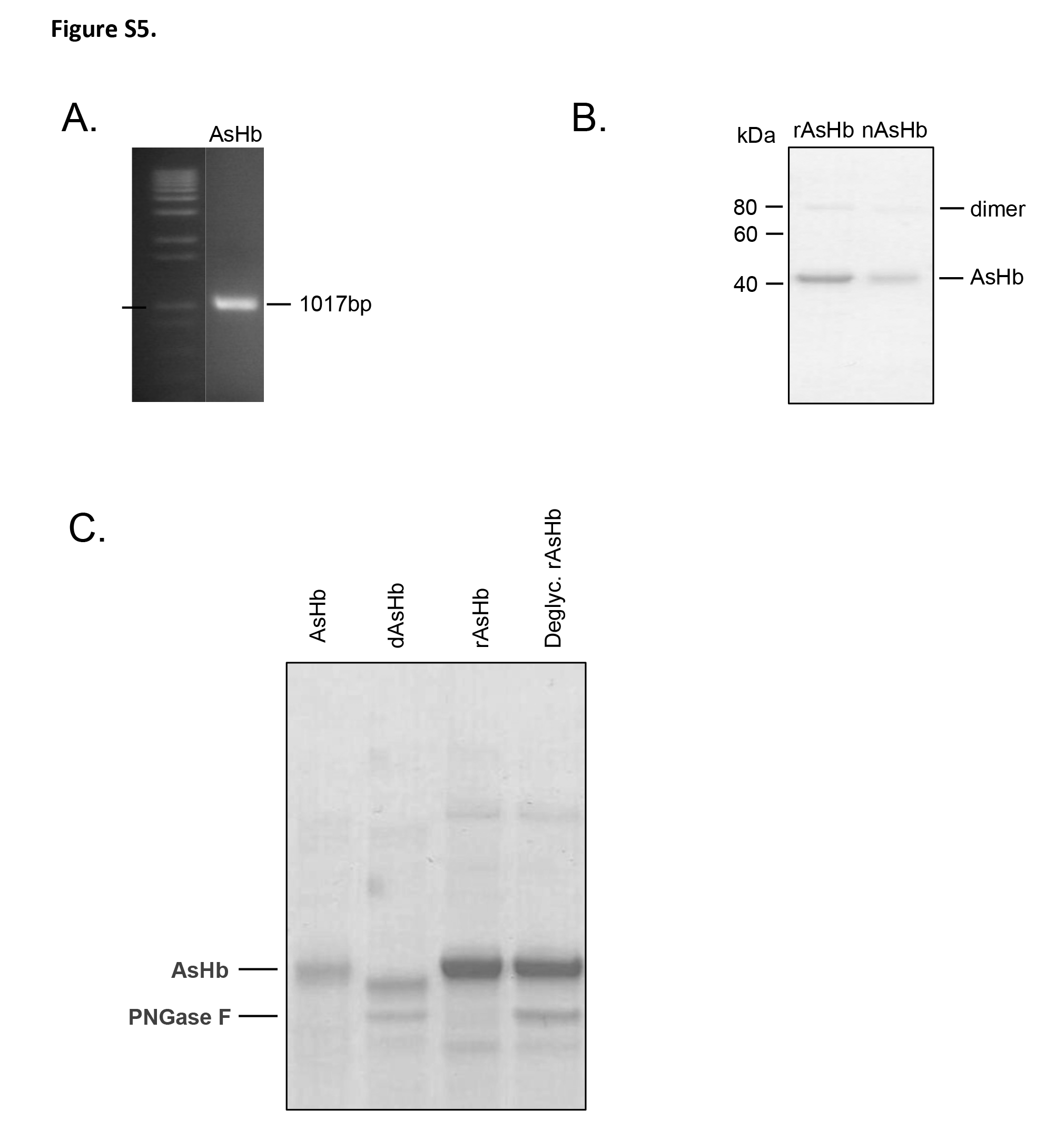

Supplement: S5 Fig — (A) AsHb cDNA was amplified from total adult A. suum cDNA. (B) The AsHb was cloned and expressed in E. coli and purified by affinity chromatography. The native (AsHb) and recombinant AsHb (rAsHb) look identical on Coomassie stained SDS-PAGE gel. (C) A Coomassie stained SDS-PAGE gel of the AsHb and rAsHb before and after deglycosylation with PNGase F. (TIF) [file pntd.0004532.s007.tif]

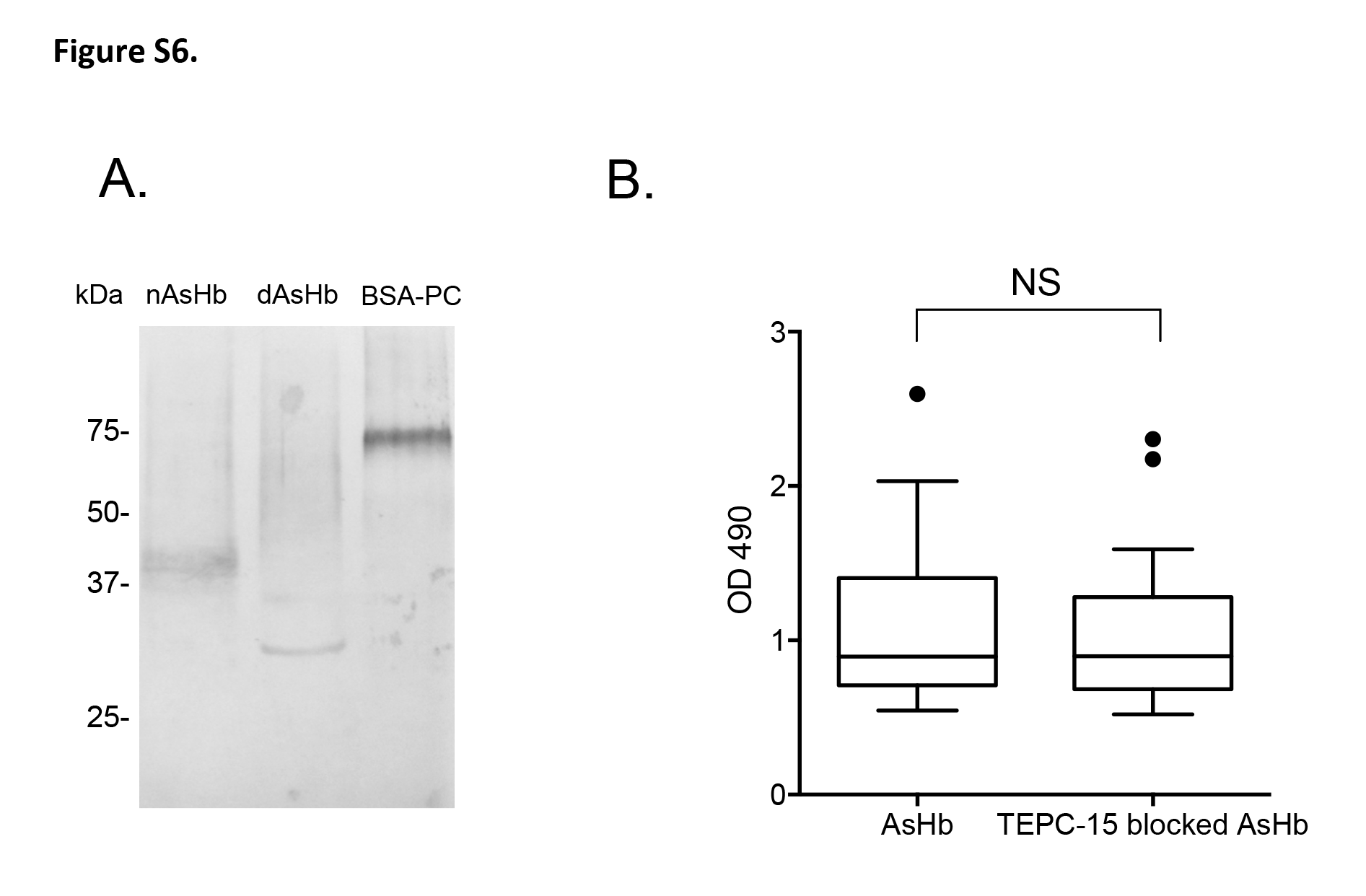

Supplement: S6 Fig — (A) The recognition on Western blot of AsHb, dAsHb and PC linked to bovine serum albumin (BSA-PC) by anti-PC monoclonal antibodies (TEPC-15). (B) A Tukey box plot representing the values of 20 A. lumbricoides positive (EPG) plasma samples shows no significant (NS) difference in the intensity of detection of AsHb and AsHb that was blocked with TEPC-15 antibodies (1:500 dilution) for 2 hours. (TIF) [file pntd.0004532.s008.tif]
